# Protracted cluster of Group A Streptococcal infection among individuals receiving wound care in the community, North East England, 2022: an outbreak report

**DOI:** 10.1017/ash.2025.44

**Published:** 2025-03-17

**Authors:** Gayle Dolan, Juliana Coelho, Yan Ryan, Angela Scott, Melanie Milburn, Chris Settle, Theresa Lamagni

**Affiliations:** 1 North East Health Protection Team, Chief Medical Advisor’s Group, UK Health Security Agency, Newcastle, UK; 2 Healthcare Associated Infection & Antimicrobial Resistance Division, UK Health Security Agency, London, UK; 3 South Tyneside and Sunderland NHS Foundation Trust, Sunderland, UK

## Abstract

**Background:**

Outbreaks of Group A Streptococcal (GAS) infection are difficult to detect in community healthcare settings and present unique challenges for infection prevention and control (IPC). We describe investigation of a cluster of GAS among individuals receiving wound care from the same community integrated care team (CIT) and associated complexities.

**Methods:**

Prospective and retrospective surveillance for cases of invasive and noninvasive GAS infection linked to the CIT was undertaken with the local NHS trust IPC team. Screening samples were requested from staff working in the CIT (n = 191) and from staff and residents (n = 73) in care home A where several cases resided. Clinical isolates were sent to the UKHSA reference laboratory for *emm* typing and whole genome sequencing (WGS).

**Results:**

Twenty-two cases were identified over a five-month period. Eighteen had isolates available for typing, 11 of which were *emm* type 108.1 and 0-2SNPs apart on WGS. Six were different *emm* types and one *emm* type 108.1 but 9-13SNPs apart from other isolates and so excluded from the investigation. No staff infected or colonized with *emm* 108.1 were identified, and no single healthcare worker had attended all cases. GAS was isolated in the room of a case resident in care home A and found to be closely genetically related to clinical isolates.

**Conclusions:**

WGS was integral in identifying outbreak cases and a multiagency approach essential to the investigation. Unfortunately, despite this no clear source or route of transmission was identified. Further research is required to determine the most effective IPC strategies for community healthcare.

## Introduction

Group A Streptococcal (GAS) infections are typically mild but on rare occasions can present as invasive infection (iGAS), resulting in severe illness. Those with wounds or receiving wound care have been shown to be particularly vulnerable to infection.^1^ Outbreaks of GAS in long-term residential care facilities have been associated with both host (eg compromised skin integrity^2,3^) and environmental (eg deficient hand hygiene^2–7^) risk factors and can often be prolonged^2,6^ resulting in significant morbidity and mortality. Outbreaks of GAS associated with the provision of home healthcare can be challenging to detect and are less well described.^8^

Registered medical practitioners in England have a statutory duty to notify the UKHSA Health Protection Team (HPT) of iGAS cases. Following notification, the HPT conduct a risk assessment in accordance with national guidance^9^ and implement actions to reduce the spread of infection. To further facilitate outbreak detection, laboratories are requested to refer all iGAS isolates, and noninvasive GAS linked to cluster investigations to the UKHSA Staphylococcus and Streptococcus Reference laboratory for *emm* typing, and whole genome sequencing (WGS).

On January 17, 2022, the UKHSA North East HPT was notified of a case of iGAS in a resident of an elderly residential care home (care home A). Two weeks later the HPT was proactively contacted by a local microbiologist to report a case of noninvasive GAS infection in a resident of the same care home, and a possible cluster of GAS. Following further assessment, two staff members and one further resident with soft tissue infections were identified by care home A. Both residents were noted to have been attended by the same community integrated care team (CIT) for wound management. The CIT is a multiagency team which operates out of a central hub to provide care in the community to individuals in their own homes and those in residential social care facilities. The team is made up of district nurses, community staff nurses, health care assistants, and community matrons working closely with physiotherapists, occupational therapists, social workers, and voluntary representatives.

A multiagency outbreak control team (OCT) was convened to review the situation and agree actions required to control the outbreak. The outbreak investigation was closed in October 2022, six months after the last identified epidemiologically linked case. This report describes the investigation, which aimed to evaluate potential routes of transmission, highlighting the complexity and implications for infection prevention and control (IPC) policy.

## Methods

### Case finding

Cases of iGAS infection were identified from routine notification to HPTs on clinical suspicion or following microbiological confirmation.

In response to the situation, microbiology, and laboratory staff at the local NHS foundation trust were also asked to undertake active surveillance, reporting cases of localized GAS infection in care homes residents or individuals receiving care from the CIT.

The hospital trust IPC team undertook a retrospective case review of GAS cases using the CIT case management system to identify further epidemiologically linked cases who had received care at the time of their diagnosis and prior to recognition of the cluster.

### Microbiological sampling and typing

Samples were collected from symptomatic individuals as part of routine clinical care.

Screening (throat and/or wound) samples were requested from:

- all staff and residents at care home A
- members of the CIT that provided direct clinical care to residents in care home A

Following the identification of further cases resident outside care home A, screening (throat and/or wound) samples were subsequently requested from the following groups with the aim of detecting any possible previously unidentified human reservoirs of infection:

- residents of care home A on the CIT workload two months after the initial screening exercise
- residents of care home B on the CIT workload
- all staff working from the same operational base (community hub) that interacted directly with the CIT

Microbiology and laboratory staff were asked to send isolates from all cases (invasive and noninvasive) to the UKHSA *Staphylococcus* and *Streptococcus* reference laboratory for *emm* typing, the internationally recognized typing scheme for Streptococcus pyogenes.^10^ Whole genome sequencing (WGS) was completed on request given the suspicion of an outbreak, to enhance the typing resolution. *Emm* typing, WGS, and trimming were undertaken as previously described.^11^

WGS reads were mapped to an internal *emm*108 reference strain using bwa (version 0.7.12). Variants were called using GATK 2.6.5 and parsed to retain high-quality SNPs (depth of coverage ≥5, AD ratio ≥0.8. Mapping Quality ≥30, ratio of reads with MQ0 to total number of reads ≤0.05). Positions that fulfilled the filtering criteria in >0.9 of the samples were joined to produce a multiple fasta format file where the sequence for each strain consists of the concatenated variants. Recombination was evaluated and hotspots removed using Gubbins.^12^

Outbreak maximum likelihood (ML) trees were constructed (RAxML software version 8.2.8, parameters –m (substitutionModel) GTRCAT –b (bootstrapRandomNumberSeed) 12345-# (numberOfRuns) 1000)). A distance matrix generated by pairwise distance analysis generated the SNP variation numbers.

### Case definitions

The following case definitions were agreed by the OCT:

**Confirmed case**—an individual with GAS/iGAS *emm* type 108.1 infection with a specimen date on or after 01 December 2021, attended by one or more members of the CIT in the seven days prior to specimen date or screened as part of the investigation and for whom the isolate was linked by WGS to the cluster.

**Possible case**—as above but where no *emm* typing or WGS available

Cases with an *emm* type other than 108.1 or that were genetically distinct (> 2SNPs apart) were excluded from the investigation.

### Exposure information

Demographic and clinical information and dates of visits made to cases by members of the CIT in the seven days prior to specimen date until 24 hours after antibiotics were administered (or until seven days post specimen date where date of antibiotic prescription was not available) were provided by the hospital trust IPC team.

CIT staff members were asked about any recent signs or symptoms of infection including sore throat and wounds.

Site visits were undertaken by the hospital trust IPC team at care home A, care home B, and the CIT community hub to review IPC measures.

### Environmental sampling

As advised by the community IPC team, environmental locations, and shared equipment at care home A and the CIT community hub were sampled, by wiping surfaces with sterile pre-moistened Polywipe screening sponges. These were sent to the UKHSA commissioned laboratory, Newcastle for culture using nonselective media under anaerobic conditions. Any suspected colonies (including GAS and MSSA (used as marker of general cleanliness of the environment)) were identified by MALDI-TOF.

## Results

### Case finding

**Figure 1.**
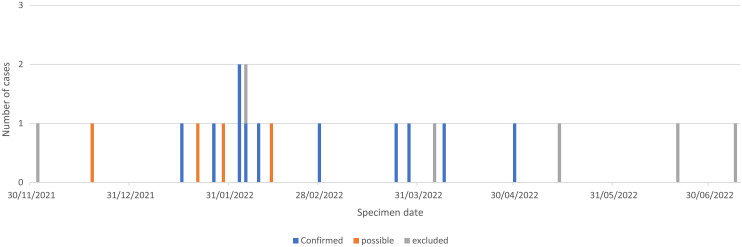
Distribution of cases in time by case definition (n = 21, specimen date not available for one asymptomatic staff member).

A total of 22 cases were identified during this investigation [table 1]. The distribution of cases in time is outlined in figure 1.

**Table 1.** Numbers screened and cases by method of identification and residential setting, North East England, 2022

Setting	No. screened	Cases (n = 22)			Confirmed cases included in investigation (n = 11)		Possible cases included in investigation (n = 4)	Total cases included in investigation
		Identified on screening	Clinical referral (GAS)	Clinical referral (iGAS)	GAS	iGAS		
Care Home A	73	3	5	1	6	1	1	8
Care Home B	14	–	2	1	1	1	1	3
Care Home C	–	–	2	–	–	–		–
Own home	–	–	3	4	–	2	2	4
CIT staff	191	1	–	–	–	–	–	–
TOTAL	278	4	12	6	7	4	4	15

Fourteen cases (6 iGAS/8 GAS) were clinically notified by the local hospital trust (specimen dates between 16/01/2022 and 08/07/2022), and four were identified following retrospective case review (specimen dates between 19/12/2021 and 13/02/2022). All 18 received care from the CIT, six in care home A, three in care home B, two in care home C, and seven in their own home.

Screening samples were requested from:

- 73 staff and residents at care home A
- 42 members of the CIT attending residents at care home A

Two samples (one resident with possible symptoms of GAS infection (buttock sore) attended by the CIT, and one asymptomatic staff member from care home A) were positive on culture for GAS.

Screening samples were subsequently requested from:

- 14 residents of care home A on the CIT workload
- 14 residents of care home B on the CIT workload
- 149 staff working in the community hub that interacted directly with the CIT

Three further samples were positive on culture for GAS; one resident of care home A with localized GAS infection (leg wound), one resident of care home B who was already a known case (previous positive wound swab), and one asymptomatic staff member within the CIT.

### Microbiological typing

Eighteen of the 22 cases had isolates available for typing and 12 of these were *emm* 108.1 (seven residents of care home A, two residents of care home B, and three individuals’ resident in their own home). The remaining six cases (two resident in care home C, two resident in their own home, one asymptomatic staff member of care home A, and one asymptomatic staff member in the CIT) were found to be different *emm* types (3 cases *emm* 12.0, 1 case *emm* 33.16, 1 case *emm* 33.0, and 1 *emm* 6.125) and excluded from the final investigation.

**Figure 2.**
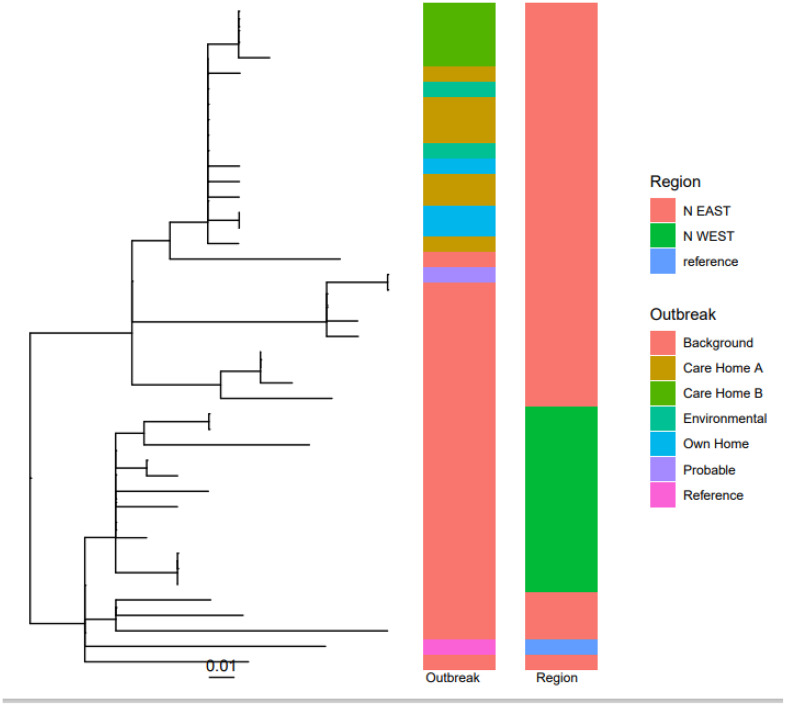
Single nucleotide polymorphism (SNP) clustering of the *emm* 108.1 whole genome sequences from isolates linked to the investigation and *emm* 108.1 background samples from the region.

All 12 *emm* 108.1 isolates underwent WGS and 11 were found to be closely genetically related (0-2SNPs apart), meeting the confirmed case definition [Figure 2]. The remaining case was genetically distinct (9-13SNPs away from other isolates) and excluded from the final investigation.

### Epidemiological investigation

Eleven confirmed and four possible cases were included in the final investigation [table 1]. Eight were residents of care home A, three residents of care home B, and four residents in their own home.

The age range of cases was 71 to 96 years (median age of 87 yr). Most cases were female (n = 11). Specimen dates ranged between 19 December 2021 and 30 April 2022.

The clinical presentation of cases included in the final investigation is outlined below [table 2]. Two cases (one possible and one confirmed case) sadly died.

**Table 2.** Clinical presentation of cases by specimen type

Clinical presentation	Specimen type		
	Blood culture	Wound swab	Throat swab
Cellulitis	2	2	–
Infected wound	–	4	–
Leg ulcer	1	3	–
Necrotic tissue	1	–	–
Pressure sore	–	1	1
**TOTAL**	4	10	1

#### Exposure information

A total of 77 visits were made by members of staff from the CIT to the 15 cases included in the investigation in the seven days prior to positive specimen, by a combination of 49 staff members [each represented by a different letter or symbol in table 3]. Twenty-one of the 49 staff members [each represented by a different colour in table 3] conducted more than one visit, 19 of which visited more than one of the 15 cases in the cluster. No single healthcare worker visited all 15 cases, and no staff member described any recent signs or symptoms of GAS infection.

**Table 3.**
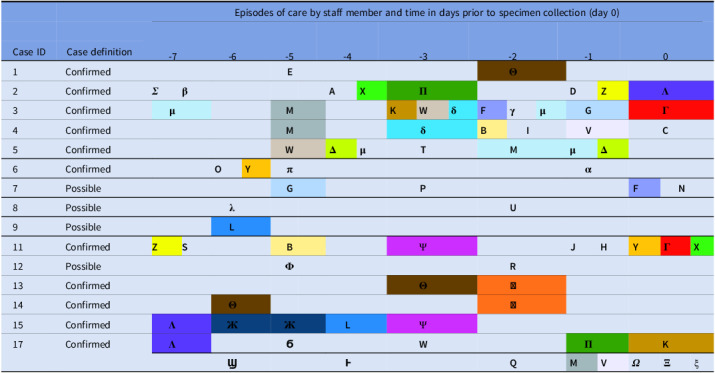
Exposure of outbreak cases (n = 15) to CIT staff in the 7 days prior to specimen date. N.B Each letter/symbol represents a unique member of staff within the CIT. Staff who delivered more than one case of patient care are highlighted in colour. Case 17 had two specimens which tested positive for GAS during the investigation and staff exposure information is provided separately for each case

Audits of hand hygiene, PPE, and bare below the elbow compliance were undertaken during site visits to the community hub. National standard infection control precautions (including use of gloves and aprons) are followed routinely by community healthcare teams and awareness of these was likely to have increased because of the focus on prevention during the COVID-19 pandemic. Some examples of missed opportunities to decontaminate hands after contact with a patient’s environment or decontaminate shared equipment after patient use and inconsistent hand washing technique were, however, observed.

Issues identified during the site visit to care home A included concerns about environmental cleanliness (including absence of a clear schedule for cleaning of soft furnishings in communal areas), and evidence of IPC policies for which the review date had expired.

No major IPC issues were identified following the site visit to care home B.

#### Environmental samples

63 environmental samples were taken at care home A on three separate occasions over a two-month period. These included samples from residents’ rooms (carpets, armchairs, sinks), communal areas (e.g., shower rooms) touch points (handrails, door handles, and pull cords) and four samples from shared equipment (blood pressure cuffs, thermometers, and hoists) [table 4].

**Table 4.** List of environmental samples from care home A

Screening session 1	Ground floor corridor chair
	Arm chair Rm 1
	Foot stool Rm 1
	Sink in Rm 1
	Door handle Rm 1
	Downstairs shower room (hoist)
	Downstairs shower room (chair and sink)
	Handrail ground floor corridor
	Lift 1st floor touch point
	Room 28 1st floor door handle
	Room 28 1st floor walking frame
	Room 28 mobile bedtable 1st floor
	Room 28 1st floor sink
	Room 28 1st floor arm chair
	Room 28 1st floor nurse call
	Handrail 1st floor corridor
	1st floor shower room 2 sink
	1st floor shower room 2 big shower chair
	1st floor shower room 2 nurse pull cord
	1st floor communal area chair
	Ground floor dining room chair
	Ground floor communal lounge chair
	Ground floor tele remote
	Downstairs shower room (chair and sink)
Screening session 2	Armchair Room 8
	Sink and tap room 8
	Table room 8
	Sink + Tap Shower 1
	Lounge chair
	Thermostat + shower
	Dining Room Table
	Dining Room Chair
	Pull Cord Shower 1
	Room 8 Door Handle
	Shower chair shower 1
	Room 8 Cream Bottle
	Room 8 Pull Cord
	Room 8 Door Handle
	Room 8 Carpet
Screening session 3	Communal BP Machine
	Ground floor shower rm 1 shower chair - communal
	Ground floor shower rm 1 communal shower head and wire
	Ground floor shower rm 1 sink communal
	Communal thermometer IP22
	Ground floor jumper communal thermometer
	Ground floor communal shower rm 1 door handle
	Ground floor corridor carpet
	Ground floor corridor hand rails
	Room 8 Door handle ground floor
	Ground floor domestic door handle
	Ground floor communal living room carpet
	Ground floor communal dining room table
	Ground floor communal dining room carpet
	1st floor communal hoist
	1st floor corridor carpet
	Stairwell carpet between floors
	Corridor handrails 1st floor communal
	Ground floor rm 8 sink area en-suite
	Rm 8 - ground floor E45 bottle
	Rm 8 - ground floor chair
	Rm 8 - ground floor carpet
	Staff room table basement
	Staff room chair basement

Two samples were positive on culture for GAS (swab of armchair and swab of carpet in the room of one of the cases). Both isolates were typed as *emm* 108.1 and found to be closely genetically related (0-2SNPs) to clinical isolates [Figure 2].

Two samples (swab of armchair and cuff of communal BP machine) were also positive for methicillin-susceptible *Staphylococcus aureus* (MSSA).

Fifty-nine environmental samples were taken at the community hub including samples from dressing bags, pool cars, keyboards, phones, carpets, door handles, and shelves in the dressing cupboard and 26 samples from shared equipment (pulse oximeters, thermometers, BP cuffs, Doppler machines, and stethoscopes) [table 5]. All were negative on culture for GAS, but two samples taken from a pulse oximeter and dressing bag in two of the pool cars were positive for MSSA.

**Table 5.** List of environmental samples from the CIT community hub

Screening session 1	Car B, Pulse Oximeter
	West store cupboard, doppler machine 1
	Car D, Thermometer
	Car F, Dressings bag
	Car A, Pulse Oximeter
	Car A, Thermometer
	West store cupboard, doppler machine 2
	Car C, BP swab
	Car D, Pulse Oximeter
	Car C, Pulse Oximeter
	Car B, Car boot cage
	Car F, Kit bag
	Car D, Red kit bag
	Car F, boot
	Dressing bag
	Car A, Dressing bag
	Car F, Thermometer
	Car C, Thermometer
	Car C, Car boot cage
	Car A, Red kit bag
	Car B, Thermometer
	Car B, Red kit bag
	Car B, Dressing bag
	Car A, BP cuff
	Car F, BP cuff
	Car D, Dressing bag
	Car D, BP Cuff
	Car D, car boot
	Car F, Pulse Oximeter
	Car B, BP cuff
	Car A, boot cage
	Manual doppler 81605
	Electrical doppler 78799
	Manual doppler 81604
	Doppler 78793
	Electrical doppler 78795
	Electrical doppler 78798
	Electrical doppler 78776
	Manual doppler 71129
Screening session 2	ACT Phone
	Sign in station
	East Phone
	Carpet dressing
	PPE Station
	Fam Team 1
	Moveable board and pens
	Photocopier
	Phone team 2
	Team 2 keyboard and mouse
	Acute care keyboard and mouse
	Team 1 keyboard and mouse
	Team 1 phone
	Photocopier
	Stethoscopes
	Tables
	Handles Kitchen
	Shelves dressing cupboard
	Kitchen handles
	East keyboard mouse

## Discussion

We identified a large and protracted cluster of GAS. Four of the cases had iGAS and two sadly died highlighting the high morbidity associated with outbreaks among vulnerable populations.

Cases were initially associated with care home A, but retrospective and prospective case finding identified others outside that setting that were epidemiologically linked to the same CIT. Although less frequently described than in other settings, previous outbreaks thought likely to be associated with provision of home healthcare have been identified.^8^ The CIT in this outbreak worked across multiple settings within the same local authority area, yet cases were only observed in two care facilities and a small number of individual homes, making it difficult to ascertain the significance of the link based on epidemiological evidence alone.

Twelve cases distributed within and out with care home A were found to be *emm* 108.1. *Emm* 108.1 was among the top 10 most frequently observed subtypes of GAS in England at the time of this outbreak^13^ and had emerged relatively recently, initially being linked to skin and soft tissue infections among prison, drug users, and homeless populations.^14^

Eleven cases were found to be closely genetically clustered (0–2 SNPs apart). Isolates linked to GAS clusters have been shown to form a distinct monophyletic clade following WGS in previous outbreak investigations,^6^ and OCT members agreed that the sequencing results strengthened the hypothesis that a common route of infection was likely. Clusters of similar isolates with and without apparent links to healthcare^15^ have been previously described. Given no other more plausible epidemiological link could be identified between cases in this outbreak, it was agreed that some form of transmission between patients and staff in the CIT was plausible. Information regarding other possible sources of exposure was, however, limited since this is not routinely recorded in community healthcare settings.

WGS data were not available until three weeks after the cluster was first recognized, and control measures were implemented in advance given the prospective detection of cases and suggestion of ongoing transmission. Staff and residents in care home A and within the CIT were treated with chemoprophylaxis, in accordance with national guidance.^9^ Some previous outbreak investigations have identified evidence of colonization among epidemiologically linked staff (although not always with the same strain as the outbreak associated strain^5^) whilst others have failed to detect any staff colonization/carriage.^3^ In this outbreak, two colonized staff members were identified but neither had the outbreak strain. Screening of staff was, however, undertaken using self-administered throat swabs, and it is possible that these may have failed to identify GAS carriage due to changes in bacterial density, poor technique, or colonization of other sites.^8^ A small number of WGS linked cases arose after completion of prophylaxis. It is possible that chemoprophylaxis may not have been 100% effective in eliminating asymptomatic colonization, or that it may not have impacted on the most likely reservoir of infection. Concerns about the effectiveness of mass chemoprophylaxis have previously been raised,^16^ and this may be less effective where there is potential for re-exposure from external reservoirs.

Information about exposure of cases to staff in the CIT was gathered to further explore the hypothesis of staff to patient transmission epidemiologically. A seven-day window prior to onset/specimen date was used to define a period of risk of acquisition of infection. No single healthcare worker had seen all 15 cases in the 7 days prior to onset, and no staff described any recent symptoms of GAS infection suggesting a single infected or colonized healthcare worker was unlikely to be acting as a reservoir of infection for all cases.

Whilst typically transmitted from person-to-person via respiratory particles, outbreaks associated with exposure to contaminated environments have also previously been described^17^ GAS was isolated in the environment of care home A and WGS demonstrated that isolates were closely genetically linked to human cases suggesting environment to person transmission was plausible. Shedding of GAS from colonized wounds has been demonstrated during care,^5^ and previous studies have shown that having an infected or colonized roommate is associated with risk of infection.^3,18^ GAS has also been found to remain in dust on furniture and equipment, and environment to person transmission via contact with contaminated surfaces and/or fomites hypothesized.^19,20^ Care home settings are not clinical environments and where environmental contamination occurs (for example within soft furnishings) it may be difficult to eradicate. Given the high proportion of cases in the cluster resident in care home A, OCT members agreed that it was likely that there had been transmission within that setting with subsequent spill over perhaps via staff or their equipment to other settings. IPC audits showed some missed opportunities to decontaminate hands and shared equipment after patient use which would provide a plausible route of transmission to other settings should contamination have occurred. GAS was not identified following screening of any shared equipment or in the environment at the CIT hub, although MSSA was isolated from some samples which may suggest sub-optimal decontamination. Some studies suggest that GAS may not survive for prolonged periods in the environment and is less hardy that other bacteria such as MRSA^21^, so it is possible that any transient reservoirs may have been missed during environmental screening exercises. The OCT concluded that undetected colonization of staff or contamination of equipment or staff may have occurred following exposure to multiple temporary environmental and/or human reservoirs of infection resulting in prolonged transmission.

WGS was fundamental to this investigation in strengthening the hypothesis that there was a likely common route of infection and in demonstrating the role for potential environment to person transmission. Despite review of IPC practices and widespread screening of staff, residents and the environment, no clear source or route of transmission was identified which would explain all cases observed. Results from WGS nevertheless helped to reinforce the importance of stringent IPC measures going forward. There are ethical and logistical challenges in implementing IPC strategies in community care settings which are not clinical environments, and further research is required to better understand the most effective control measures.^22^
